# Dual ensemble system for polyp segmentation with submodels adaptive selection ensemble

**DOI:** 10.1038/s41598-024-56264-2

**Published:** 2024-03-14

**Authors:** Cun Xu, Kefeng Fan, Wei Mo, Xuguang Cao, Kaijie Jiao

**Affiliations:** 1 Guilin University of Electronic Technology, Guilin, 541000 China; 2 China Electronics Standardization Institute, Beijing, 100007 China

**Keywords:** Computational models, Image processing, Computational models, Image processing

## Abstract

Colonoscopy is one of the main methods to detect colon polyps, and its detection is widely used to prevent and diagnose colon cancer. With the rapid development of computer vision, deep learning-based semantic segmentation methods for colon polyps have been widely researched. However, the accuracy and stability of some methods in colon polyp segmentation tasks show potential for further improvement. In addition, the issue of selecting appropriate sub-models in ensemble learning for the colon polyp segmentation task still needs to be explored. In order to solve the above problems, we first implement the utilization of multi-complementary high-level semantic features through the Multi-Head Control Ensemble. Then, to solve the sub-model selection problem in training, we propose SDBH-PSO Ensemble for sub-model selection and optimization of ensemble weights for different datasets. The experiments were conducted on the public datasets CVC-ClinicDB, Kvasir, CVC-ColonDB, ETIS-LaribPolypDB and PolypGen. The results show that the DET-Former, constructed based on the Multi-Head Control Ensemble and the SDBH-PSO Ensemble, consistently provides improved accuracy across different datasets. Among them, the Multi-Head Control Ensemble demonstrated superior feature fusion capability in the experiments, and the SDBH-PSO Ensemble demonstrated excellent sub-model selection capability. The sub-model selection capabilities of the SDBH-PSO Ensemble will continue to have significant reference value and practical utility as deep learning networks evolve.

## Introduction

The third most common form of cancer worldwide is colorectal cancer, and its prevalence is increasing every year^1^. About the precursors of colon cancer, it is commonly accepted that most colorectal cancers evolve from adenomatous polyps^2^. Recent surveys and statistics underline polypoid lesions are precursors to most ( 85%) colorectal cancers^3^. Colonoscopy is the ‘gold standard’ method for examining colon and rectum^4,5^. Importantly, it has been assessed that the proportion of colon polyps missing during endoscopies could range from 20 to 47 percent^6^. A review article noted that an early detection of the CRC increases the 5-year survival rate from 18% when CRC is detected in the highest grade to 88.5% when it is detected in an initial grade due to symptoms^7^. Along with the development of artificial intelligence, semantic segmentation methods of AI assisted colonoscopy detection can significantly reduce the risk of misclassification and omission of polyp cancer, colorectal tumor lesions and colorectal cancer from early to late stages due to various reasons^8^. Therefore, the accuracy of semantic segmentation of colon polyps needs to be improved to achieve better support for colonoscopy detection.

**Figure 1:**
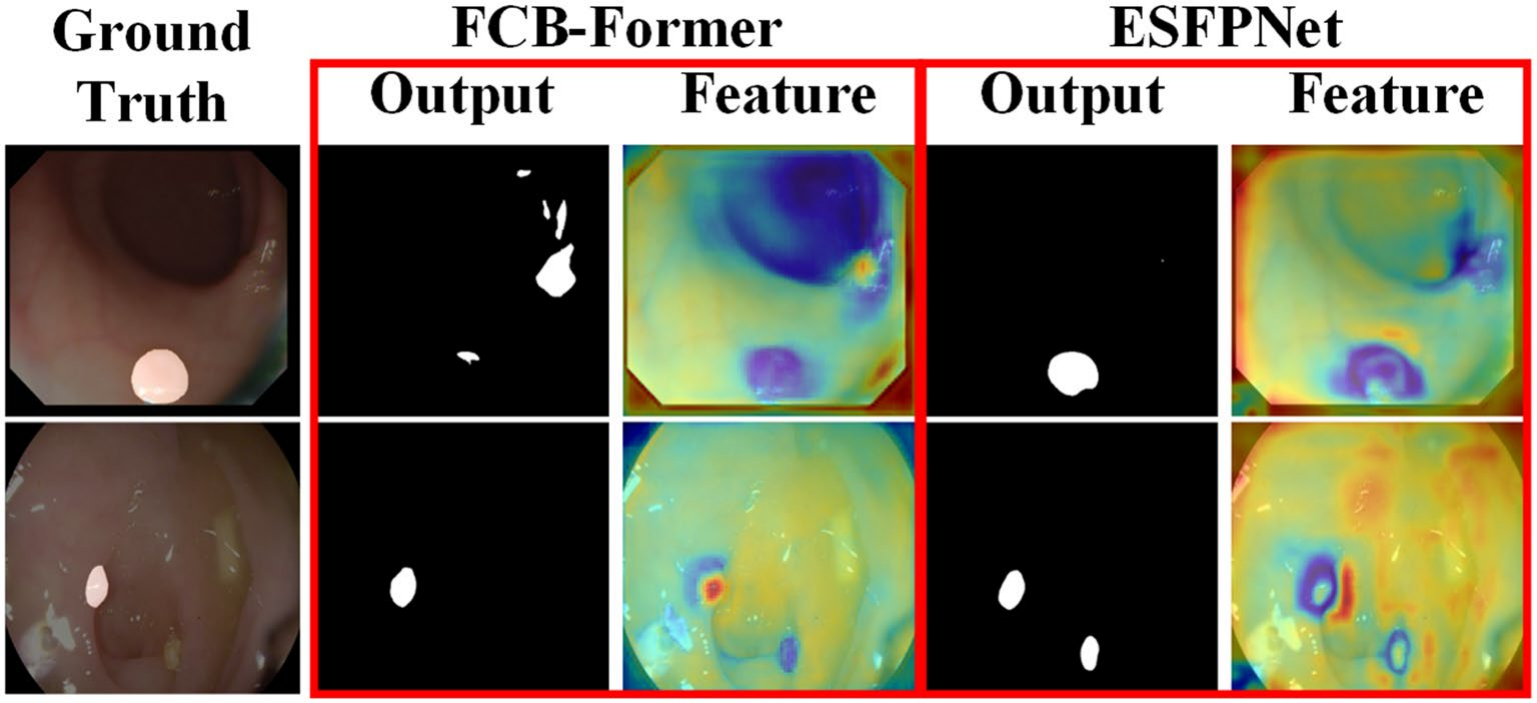
Feature heat maps and polyp segmentation results under CVC-ColonDB and ETIS-LaribPolypDB datasets.

Many networks for deep learning have achieved advanced performance in polyp-by-pixel segmentation^9^. The backbone of many of these excellent networks is the Pyramid Vision Transformer V2 (PVTv2)^10^ or the Mixed Transformer (MiT)^11^. High-level semantic features are more appropriate for the model to achieve a higher performance^12^. Feature fusion, a common technique in polyp segmentation tasks, has shown exceptional results^13,14^. However, there are still advanced semantic features that can further improve the segmentation accuracy through feature fusion. The process of discovery is to select the location of the feature maps according to Di et al^15^. and refer to Han WC et al^16^. to generate the feature maps of FCB-Former^17^ and ESFPNet^18^ in the form of heat maps, as shown in Fig. 1. The darker the warm color on the feature map indicates the more obvious features of the polyp or the background, while there is a clear change from warm to cold color at the polyp-background junction. It can be found that the features are not complete enough to lead to accurate segmentation results. In order to solve this problem, we propose a fusion strategy, Multi-Head Control Ensemble, which fuses complementary features step-by-step and integrates different feature results optimally to achieve efficient utilization of complementary features.

Colon polyps are thought to vary widely in size, orientation, color and texture^19^. It is difficult for a single network to produce accurate predictions in various situations^20^. Employing a multi-network ensemble strategy is anticipated to both enhance and stabilize performance. However, variability among polyps, coupled with the risk of networks converging to a local optimum during training, may result in a network that adversely affects the effectiveness of the ensemble at the end of training. The proactive identification and removal of such a network before the conclusion of training represent a challenge. A review article points out that generalisability studies are very limited in medical image analysis^21^. To solve the above problems, we propose a generalisability ensemble learning strategy that adaptively selects the most suitable network for the ensemble for different datasets, thus stabilizing the output of high-performance segmentation results.

The main contributions of this paper are as follows:

1. In order to maximize the use of complementary features, we propose Multi-Head Control Ensemble (MHC Ensemble), which can effectively supervise the network and output high-precision segmentation results.
2. In order to achieve stable and high-performance segmentation on discrepant data, we propose an improved Particle Swarm Optimization algorithm for optimizing sub-model weights in ensemble learning. And based on this, we propose a strategy SDBH-PSO Ensemble that can perform adaptive selection of sub-models under different datasets.

## Related work

### Ensemble learning

Ensemble learning methods are broadly categorized into: bagging, boosting, and stacking^22^. Bagging ensemble improves accuracy by training a single network with multiple copies of the dataset^23^. Boosting ensemble optimizes the ensemble results by assigning greater weights to the erroneous copies on top of the bagging ensemble by assigning greater weights to the erroneous copies to optimize the integration results^24^. Combining multiple models helps to improve and stabilize the results^25^. Stacking ensemble’s approach of integrating multiple networks as sub-models can provide strong robustness^26^. Stacking ensemble and ensemble multi-output is expected to solve the task of colon polyp segmentation, which is difficult for a single network. For the sake of simplicity, we will refer to “ensemble multi-output” as “ensemble” elsewhere in this paper.

Kang et al^27^. used ensemble learning to ensemble segmentation results from Mask R-CNN networks using ResNet50 and ResNet101 as the backbone. Thanh et al^28^. used ensemble learning to ensemble UNet segmentation results from EfficientNet B4 and EfficientNet B5. Nanni et al^29^. used the PvTv2 segmentation ensemble on other tasks and achieved excellent segmentation results. The sub-models used for the ensemble have changed as the model performance has improved. A review article on ensemble learning points out that a major challenge in deep ensemble learning is model selection for building the ensemble architecture^30^.

In the model selection problem, Zhang et al^31^. used neighborhood mutual information to select the models involved in the ensemble on carbon emission prediction. Djellali et al^32^. selected the models involved in the ensemble in a data mining task based on k-fold cross-validation. Both of the above methods perform sub-model selection at the end of training. Birman et al^33^. used reinforcement learning for sub-model selection during training in malware detection tasks. Labeling for colon polyp segmentation is more expensive, which limits the application of reinforcement learning in this area. To explore the application of model selection to colon polyp segmentation, we propose the SDBH-PSO Ensemble.

### Ensemble learning with improved PSO optimization

The most critical aspect of the ensemble is the optimization of the ensemble weights and the selection of sub-models, and the Particle Swarm Optimization (PSO) algorithm^34^ is commonly used for the optimization of the ensemble weights^35^. PSO algorithms have been used to solve a variety of mathematical, engineering, design, network, robotics, and image processing optimization problems^36^. The solution in the PSO algorithm is represented as a particle, which holds a position vector and a velocity vector. PSO searches for the optimal solution by iteration. In each iteration, the velocity $v_{id}(t)$ of each particle is updated based on its previous optimal position $P_{pd}(t)$, the current optimal position $P_{gd}(t)$ of all particles, random numbers $r_1$ and $r_2$ in [0, 1], adjustable inertia parameter $\omega$, and adjustable learning parameter $c_1$ and $c_2$, while the position $x_{id}(t)$ is varied as the velocity changes, as defined in Eqs. (1) and Eqs. (2).

1 $$\begin{aligned} v_{id}(t+1)&={\omega v}_{id}(t)+c_1r_1(P_{pd}(t)-x_{id}(t))+c_2r_2(P_{gd}(t)-x_{id}(t)) \end{aligned}$$

2 $$\begin{aligned} x_{id}(t+1)&=x_{id}(t)+v_{id}(t+1) \end{aligned}$$

The PSO algorithm is considered to continue to be dynamic in interdisciplinary research in the future^37^. In recent years, Gu et al^38^. proposed a resampling PSO algorithm for optimizing the scheduling of multi-star, large-area target observations. Subsequently, Song et al^39^. proposed a large-scale nonconvex joint optimization method based on PSO in order to solve the active control problem of wind farm layout and turbine yaw. Fontes et al^40^. proposed an improved PSO algorithm based on the job shop scheduling problem of transportation resources to be solved. Similarly, Qian et al^41^. proposed an improved PSO algorithm, which successfully realized the intelligent selection of the piston sealing groove for the designed domestic cylinder. Du et al^42^. proposed an improved PSO algorithm for ordered charging strategy, which can reduce the charging cost and peak variance of electric vehicles. Thus, on the problems that can be optimized by PSO algorithms, designing and improving PSO algorithms based on the problem to be solved or optimized is expected to solve the problem in a better way.

### Image segmentation on colon polyps

On the task of semantic segmentation of colon polyps, this paper focuses on realizing high-precision and stable segmentation of polyps by building branches and feature fusion, and the relevant state of the art in this regard is as follows.

On branch building, Guo et al^43^. proposed a two-branch approach called ThresholdNet to collaborate segmentation and threshold learning in alternative training strategies. Fang et al^44^. proposed a new boundary-sensitive loss to model the interdependence between region branches and boundary branches. In order to better extracte the detail information, Zhang WC et al^45^. used to capture the local appearance details through the dual branch structure of Transformer and CNN. Chen et al^46^. built a depth feature extraction branch and depth bootstrapping for extracting the depth information between pixels. Wang et al^47^. built a new anchor-free instance segmentation framework by performing object detection branching for classification and localization with mask generation branching for generating instance-level masks. Fan et al^48^. achieved a more stable training process in federated learning by building a multi-branch network.

For feature fusion, Huang et al^49^. re-weighted encoder features in space and channel to enhance key features for segmentation task. To enhance the features on the boundary, Zhou et al^50^. merged the boundary information into the segmentation network to generate finer segmentation maps. Liu et al^51^. achieved adaptive feature fusion and selection for the network by channel attention. In addition, Chen et al^52^. utilized rich global context information to refine the fused features for informative feature representation. Patel et al^53^. improved the quality of features layer by layer, which in turn enhanced the final feature representation. Wang et al^54^. suggested that the region around the polyp has more detailed features that facilitate polyp segmentation.

## Method

### Overview

**Figure 2:**
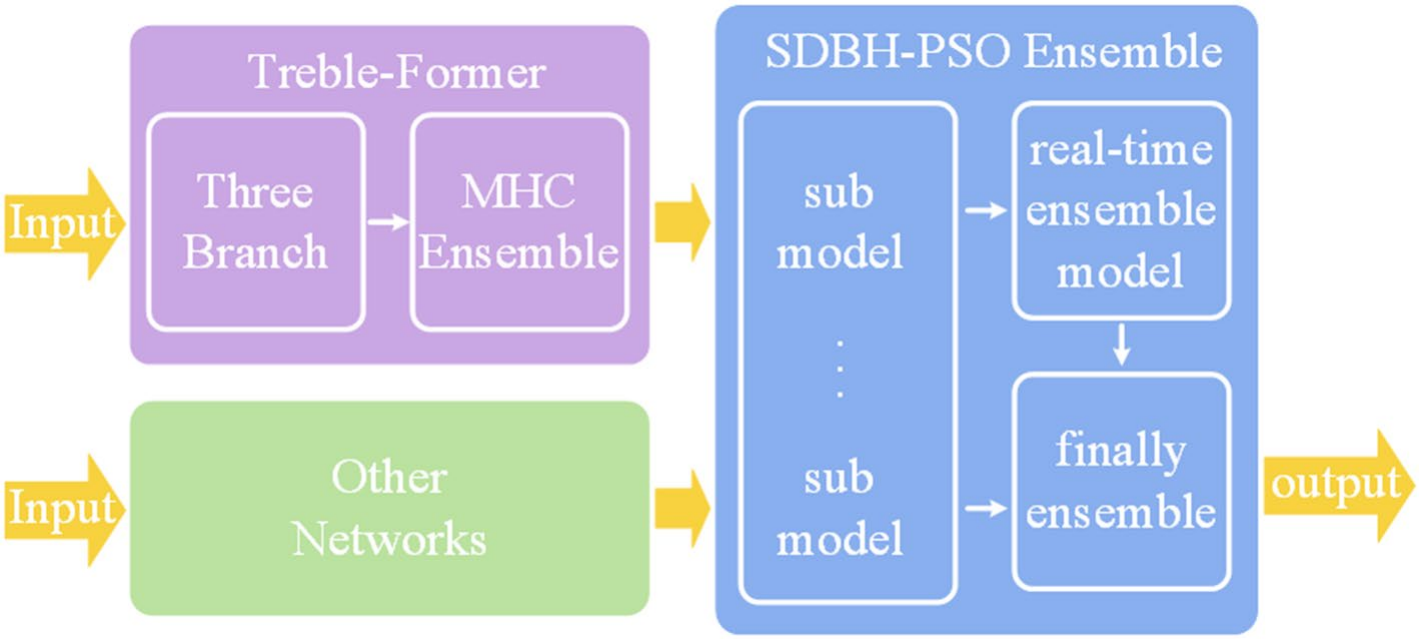
The architecture of Dual Ensemble System with Treble Transformer.

In order to fuse complementary features and perform stable high-performance segmentation on the disparate colon polyp dataset, we built the Dual Ensemble System, as shown in Fig. 2. Among them, in order to provide complementary features, we built Three-branch Architecture, which fuses complementary features through MHC Ensemble. In addition, in order to achieve stable and high-precision segmentation on different datasets, we choose FCB-Former and ESFPNet, which have complementary phenomena in the output results, and also take into account that there are differences in the adaptability of different datasets to the optimal network depth. The sub-models selected for the SDBH-PSO Ensemble range from sub-model 1 to sub-model 6 and include the following: Treble-Former-L(MiT-B4, PvTv2-B4), Treble-Former-S(MiT-B2, PvTv2-B2), FCB-Former-L(PvTv2-B4), FCB-Former-S(PvTv2-B2), ESFPNet-L(MiT-B4), ESFPNet-S(MiT-B2). Finally, the best real-time ensemble model and the best sub-model optimized by SDBH-PSO are again subjected to final ensemble. In addition, DET-Former is an ensemble structure that allows segmentation across multiple devices. It has an FPS of 3.9 for single-image input.

### Three-branch architecture

**Figure 3:**
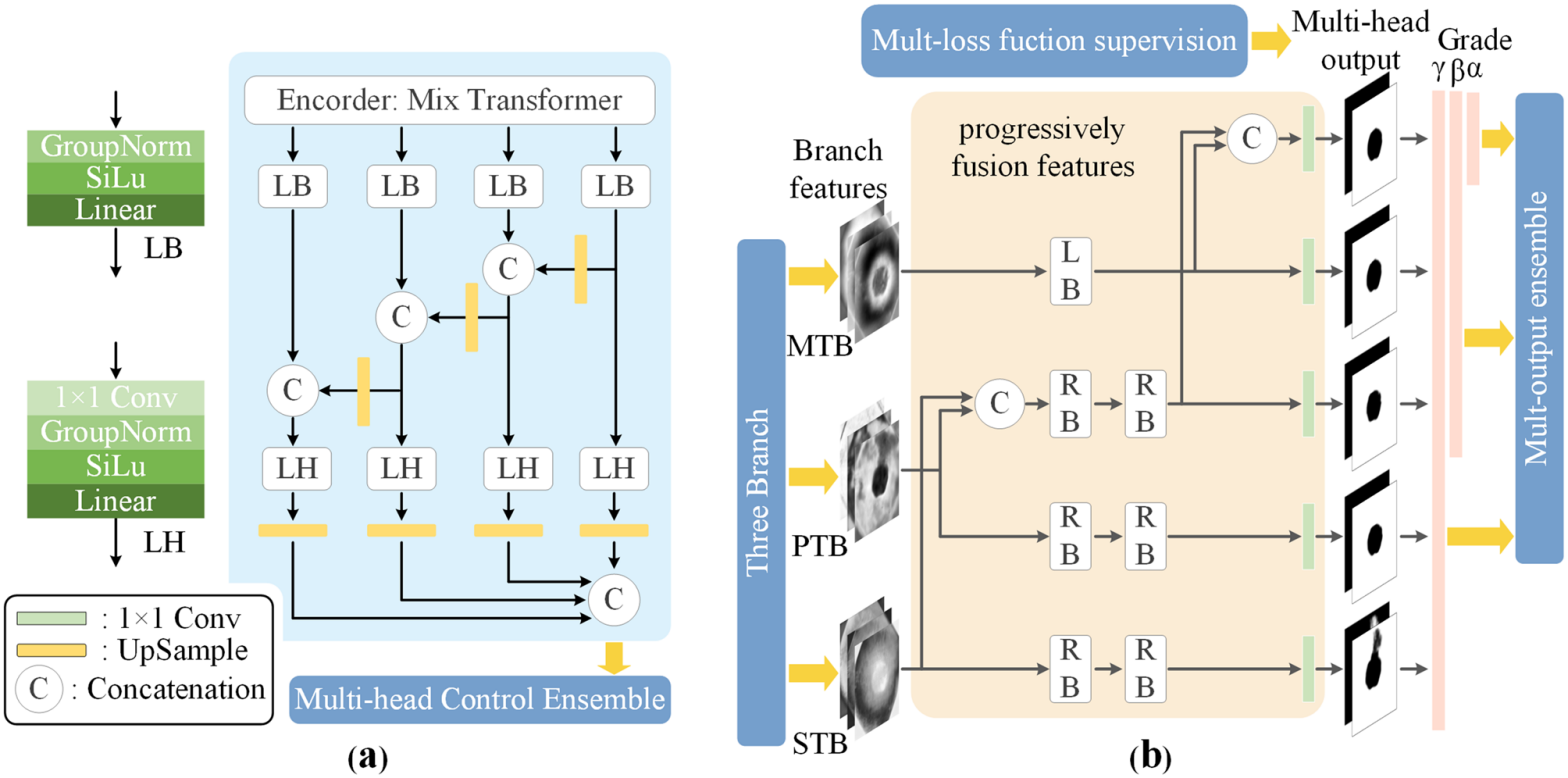
(**a**) The architecture of Mix Transformer Branch. (**b**) The architecture of Multi-Head Control Ensemble.

**Mix Transformer Branch (MTB).** In order to stabilize the performance during training so as to facilitate the integration with other branches, we constructed the MTB as shown in Fig. 3a. In order to improve the consistency of the convergence speed of the training parameters in each branch of Treble-Former, we add GroupNormal as a normal layer before the linear layer, which can stabilize the performance and reduce the effect of batch size on the model, and ultimately make it easier for MTB to integrate with the other branches to become a powerful network. In addition, both polyp and background features should be concerned in polyp segmentation. Therefore, we use SiLu as the activation function, which can better preserve both polyp and background features in each feature map.

**Pyramid transformer branch (PTB).** In order to maintain the complementarity of the features in Fig. 1, we retain some of the structures in FCB-Former. Since the Transformer Branch in FCB-Former uses PVTv2 as the backbone, and PVTv2 uses convolutional layers instead of the linear layers of the traditional Transformer, PVTv2 is able to capture the information of the polyp boundaries when sensing the global field of view as well as CNN. So we remove the full convolutional branch of FCB-Former and keep the Transformer Branch as the PTB in Treble-Former.

**Swin transformer branch (STB).** In order to make the STB output different features from the first two branches, we adopt DoubleUNet^55^ as the structure of the STB. DoubleUNet has good feature fusion capability on a network with UNet as the encoder. Since Swin Transformer^56^ does not use a convolutional layer, the improvement of the extraction ability of features on the details of colon polyps can be realized by combining VGG19 with a stacked 3$\times$3 convolution. Therefore, we fused the SwinUNetR^57^ equipped with Swin Transformer with the UNet equipped with VGG19 for coarse and fine features by using the structure of DoubleUNet.

### Multi-head control ensemble

**Multi-head control ensemble (MHC Ensemble).** As shown in Fig. 3b, three branches output branching features. In order to fuse the complementary branch features, the branch outputs are cascaded step by step through the RB module and LB module of FCB-Former. In addition, multi-loss function supervises and multi-head output Ensemble are also performed on the results of multi-head output, and this whole process is collectively called Multi-Head Control Ensemble.

**Multi-loss function supervises.** In the problem of binary classification of polyp and background, we expect the deep model to learn the polyp and background features while paying more attention to the representative features of the polyp. Therefore, we then chose the combination of the cross entropy loss function (CE loss), which pays attention to the background and polyps, and the *Dice* loss function, which pays attention to the polyps only, as the loss function supervised training for each output header.

**Multi-head output Ensemble.** In order to complement the output results in the multi-head output, we first empirically categorize the five multi-head outputs in Fig. 3b into three categories according to performance from highest to lowest: ($\alpha$) MTB concatenated PTB concatenated STB’s output head; ($\beta$) MTB’s output header and the output header after PTB concatenate STB; and ($\gamma$) the output header of PTB and STB. In order to take into account, the performance differences of each output head in a specific case, when integrating the output results of multiple output heads, the definition of weights will be based on the specific division of weights according to the *mDice* coefficients, the evaluation indexes of each output head on the validation set.

3 $$\begin{aligned} W_{head}&=\textstyle \sum _{i}^{I}Softmax(d_j) \end{aligned}$$

4 $$\begin{aligned} Output_{Ensemble}&=\textstyle \sum _{i}^{I}(Output_{head\ i}\cdot W_{head\ i}) \end{aligned}$$

where $J\in {\left\{ \alpha ,\ (\alpha +\beta ),(\alpha +\beta +\gamma ) \right\} }$. *d* denotes the evaluation index *mDice* corresponding to the corresponding output head. $Output_{head\ i}$ denotes the output result of the output head. The *mDice* coefficients corresponding to each class in *J* are Softmaxed and then accumulated to generate the ensemble weights $W_{head}$. The weights are weighted and summed with the outputs of the header $Output_{head\ i}$ to generate the integrated prediction result $Output_{Ens}$.

### SDBH-PSO

Since the global optimal solution before iteration in the real-time ensemble task is not necessarily the global optimal solution in this epochs, there is a need to prevent the optimal particle from being a local optimal solution. Therefore, it is necessary to initialize the particles that are too close to the optimal particles when the whole is too aggregated. The degree of proximity of each particle to the optimal particle is defined by Pearson’s correlation coefficient^58^, and the overall aggregation of particles is defined by Renxoa Wan’s aggregation coefficient *C*(*k*)^59^.

5 $$\begin{aligned} {sim}_i&=\frac{Cov(X_{id},P_{gd})}{\sqrt{Var(X_{id})}\sqrt{Var(P_{gd})}} \end{aligned}$$

6 $$\begin{aligned} C(k)&=\sum _{j=1}^{n}{\frac{{sim}_j}{\sum _{i=1}^{n}{sim}_i}{sim}_j} \end{aligned}$$

where *C*(*k*) is the aggregation degree of the particle population in the kth generation and n is the population size. Since the iterations are all relatively homogeneous and may lead to excessive oscillations in particle aggregation in later iterations, an adaptive function $\theta (k)$ controlled by a nonlinear function is added to assess the degree of particle overlap^59^. Whether the particles have a tendency to fall into local optimum is judged by $H \cdot \theta (k)<C(k) \cdot {sim}_j$, where H is a constant used for adjustment. Through many experiments, it is found that SDBH-PSO has the best effect on weight adjustment when H is taken around 3.

7 $$\begin{aligned} \theta (k)={\left( \frac{k_{max}-k}{k_{max}}\right) }^s \cdot (\lambda _{max}-\lambda _{min})+\lambda _{min} \end{aligned}$$

Where $k_{max}$ is the maximum number of iterations and *k* is the current number of iterations. *s* is an exponential factor. We set $s=1.0$, $\lambda _{max}=0.9$, $\lambda _{min}=0.4$ in our experiments, while the adjustable inertia parameter in Eqs. (1) is also set to $0.9-0.5{({k}\setminus {k_{max}})}^2$ with reference to the nonlinear tuning method.

The potential global optimal point is usually within a certain distance from the current optimal point, and we found that in our task, the variation of the distance between the previous generation global optimal point and the next generation is roughly concentrated in the range of [0.04, 0.23] through analysis. The strategy of RBH-PSO^60^ to search for the potential global optimum is used to randomly select a point within a certain range as the location of the potential optimum ${\widetilde{x}}_{id}$. Compared to the RBH-PSO in which the radius $\zeta =0.01$ is taken as the range, the global optimum solution of the real-time ensemble is subjected to the influence of the model training and has a large variation. So, the search for potential optimal solutions needs to be expanded. Therefore, the position of the particle to be reset is placed into the black hole combined with randomizing the initial velocity to ${\widetilde{v}}_{id_0}$ for resetting.

8 $$\begin{aligned} {\widetilde{x}}_{id}(t+1)&=P_{gd}+\zeta \end{aligned}$$

9 $$\begin{aligned} {\widetilde{v}}_{id_0}&=(v_{id_{max}}-v_{id_{min}}) \cdot P_{gd} \cdot rand \cdot Gaussian(\mu ,\sigma ^2) \end{aligned}$$

10 $$\begin{aligned} \sigma&=(\sigma _{max}-\sigma _{min}) \cdot \frac{k}{K} \end{aligned}$$

where $\zeta$ is randomly derived from a uniform distribution over the interval $[-\xi , \xi ]$, and $\xi$ is taken as 0.1. *Rand* is a random number in the range [0, 1], $x_{id\ max}$ and $x_{id\ min}$ are the upper and lower bounds of the search space, and $Gaussian(\mu ,\sigma ^2)$ is a Gaussian function. We set $\sigma _{max}=1.0$ and $\sigma _{min}=0.1$.

Similarity degree black hole PSO can be summarized simply in Algorithm 1.

**Algorithm 1:**
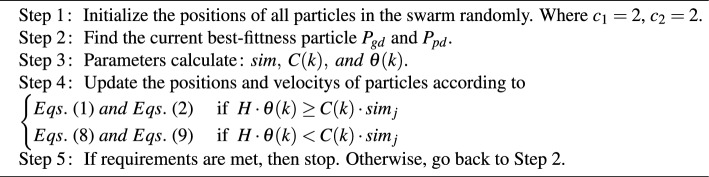
**SDBH-PSO algorithm**

### SDBH-PSO ensemble

We use the ensemble weights as the positions of the particles in SDBH-PSO, and the *mDice* coefficients achieved by the Ensemble Learning outputs as the fitness of the particles in the validation set, and achieve the optimal allocation of the ensemble weights to the validation set through the SDBH-PSO algorithm: every ten epochs. In addition, the initial learning rate is 0.001, and the learning rate is adjusted with the strategy that the learning rate decreases to half if the *Dice* coefficient has not improved for five consecutive generations on the validation set. When the learning rate of all sub-models has decreased an equal number of times, the weights of Ensemble Learning are optimized by the SDBH-PSO Ensemble algorithm. The sub-models with weights less than or equal to 0 are marked. When the sub-model has been labeled 3 times, the learning rate corresponding to the learning rate adjustment strategy is already very low, so even if we continue to train this sub-model, it will not improve much, so we choose to eliminate it.

Regarding parameter configurations, the SDBH-PSO Ensemble performs five iterations for every ten epochs, utilizing 50 particles per iteration. Where the iterative process is the same. The running speed of the SDBH-PSO Ensemble, which optimizes parameters via validation sets, is contingent upon the size of this set. Specifically, for a validation set of 100 images, the iteration time per generation is approximately one minute. Conversely, if the validation set contains 61 images, the iteration time is reduced to around 35 s.

In contrast to other semantic segmentation that Ensemble Learning performs Ensemble by best sub-model, SDBH-PSO Ensemble performs real-time ensemble every ten epochs during the training process. The SDBH-PSO Ensemble’s best ensemble model’s checkpoint of the SDBH-PSO Ensemble is not necessarily the checkpoint of the best sub-model, we perform the final ensemble of the best ensemble model with the best sub-model, and the final ensemble is defined as follows.

11 $$\begin{aligned} L_{DET-Former}=W_{rt} \cdot L_{rt}+ {\textstyle \sum _{i}^{\widetilde{I}}}({W_{{sub}_i} \cdot L_{{sub}_i}}) \end{aligned}$$

where $L_{rt}$ is the output of the best real-time ensemble model, $L_{{sub}_i}$ is the output of the best sub-model, $W_{rt}$ is the weight hyperparameter of the best real-time ensemble model, $W_{rt}=0.5$, $W_{{sub}_i}$ is the weight hyperparameter of each best sub-model, $W_{{sub}_i}=0.5\setminus \widetilde{I}$, and $\widetilde{I}$ is the weight hyperparameter of each sub-model filtered by SDBH-PSO according to different data sets. $L_{DET-Former}$ is the final output of SDBH-PSO Ensemble.

## Experiments

### Dataset

The following datasets are used in this paper: the Kvasir^61^, CVC-ClinicDB^62^, CVC-ColonDB^63^, ETIS-LaribPolypDB^1^ and PolypGen^64^. The PolypGen comes from 6 unique centers suitable for Generalisation testing compared to other datasets. The information of the datasets is shown in Table 1. Due to the different image sizes of different datasets, we scale all the sizes to $352\times 352$ and set the batch size to 4. In this paper, we combine ESFPNet, SS-Former, and an analytical paper illustrating the effect of polyp segmentation dataset enhancement on segmentation^66^, and choose random flip, scale, rotate, as well as random expansion and erosion as the data augmentation operations.

**Table 1:** The most commonly used public dataset for polyp segmentation.

Dataset	Kvasir	ClinicDB	ColonDB	ETIS-LaribPolypDB	PolypGen
Images	1000	612	380	196	1537
Object of ratio	0.79–62.13%	0.34–45.88%	0.30–63.15%	0.11–29.05%	_
Input size	Variable	$384\times 288$	$574\times 500$	$1225\times 966$	Variable

### Evaluation metrics

Almost all of the colon polyp segmentation papers adopt the *mDice* coefficient and the *mIoU* coefficient as the evaluation performance metrics to measure segmentation accuracy. Furthermore, we choose the 95th percentile of the asymmetric Hausdorff distance (*HD*95) as a performance metric for the boundary of interest. *mDice*, *mIoU* and *HD*95 are calculated using the following formulae$:$

12 $$\begin{aligned} mDice&=\frac{1}{2}\sum _{i}^{k\in (P,B)}\frac{{2\times n}_{ii}}{\sum _{j}^{k}{n_{ij}+\sum _{j}^{k}n_{ji}}} \end{aligned}$$

13 $$\begin{aligned} mIoU&=\frac{1}{2}\sum _{i}^{k\in (P,B)}\frac{n_{ii}}{\sum _{j}^{k}{n_{ij}+\sum _{j}^{k}{n_{ji}-n_{ii}}}} \end{aligned}$$

14 $$\begin{aligned} HD95&=\max _{k95\%}[d(X,Y),d(Y,X)] \end{aligned}$$

where $n_{ii}$ denotes the number of real numbers and is predicted to be *j* and *k* is the category of polyp and background (polyp abbreviated as *P* and background abbreviated as *B*). $n_{ii}$ is the number of correctly predicted values, and $n_{ij}$ and $n_{ji}$ denote the false positives and false negatives respectively. The one-way Hausdorff distances *d*(*X*, *Y*) measure how far the predicted results are from the actual results and *d*(*Y*, *X*) as well as vice versa.

Regarding the evaluation of the success of polyp categorization without calculating the background, we choose *Dice*, which is formulated as follows$:$

15 $$\begin{aligned} Dice=\frac{{2\times n}_{PP}}{\sum _{j}^{k}{n_{Pj}+\sum _{j}^{k}n_{jP}}} \end{aligned}$$

### Compare experiment

In the compare experiment of DET-Former, we use UNet, UperNet and DoubleUNet as the base networks and SS-Former, FCB-Former, ESFPNet, HarDNet-DFUS^65^ amd Nanni’s Ens (Nanni et al^67^. proposed Ens1) as the comparison models. Experiments were conducted on five datasets: Kvasir, CVC-ClinicDB, CVC-ColonDB, ETIS-LaribPolypDB, and PolypGen. Each model was trained for 100 epochs, and the optimal values of the evaluation metrics were documented. The metrics of each model that differ most from DET-Former are taken for t-test statistical analysis and the *p*-value is generated. The results of tests using different datasets or data sources were more closely aligned with clinical scenarios and were selected to generate visual segmentation maps.

**Table 2:** The test results of the compare study in Kvasir and CVC-ClinicDB.

Train/Val	Kvasir 80%/10%				CVC-ClinicDB 80%/10%			
Test	Kvasir 10%				CVC-ClinicDB 10%			
Metric	Dice	mDice	mIoU	*p*	Dice	mDice	mIoU	*p*
UNet	0.814	0.857	0.822	< 0.05	0.871	0.87	0.876	< 0.05
UperNet	0.855	0.884	0.861	< 0.05	0.882	0.913	0.886	< 0.05
Double-UNet	0.794	0.824	0.797	< 0.05	0.832	0.841	0.841	< 0.05
SS-Former	0.898	0.940	0.896	< 0.05	0.923	0.958	0.929	< 0.05
FCB-Former	0.901	0.942	0.904	0.15	0.944	0.969	0.945	0.26
ESFPNet	0.896	0.938	0.898	< 0.05	0.945	0.971	0.946	0.41
Nanni’s Ens	0.916	0.948	0.912	0.35	0.946	0.973	0.949	0.69
HarDNet-DFUS	0.908	0.943	0.905	0.18	0.943	0.969	0.944	0.21
DET-Former	0.923	0.954	0.924	_	0.953	0.975	0.953	_

**Table 3:** The test results of the compare study in ETIS-LaribPolypDB and CVC-ColonDB.

Train/Val	Kvasir and CVC-ClinicDB 90%/10%							
Test	ETIS-LaribPolypDB				CVC-ColonDB			
Metric	Dice	mDice	mIoU	*p*	Dice	mDice	mIoU	*p*
UNet	0.668	0.798	0.741	< 0.05	0.704	0.842	0.775	< 0.05
UperNet	0.671	0.804	0.774	< 0.05	0.714	0.833	0.786	< 0.05
Double-UNet	0.633	0.795	0.760	< 0.05	0.631	0.801	0.743	< 0.05
SS-Former	0.802	0.894	0.856	< 0.05	0.789	0.882	0.836	0.18
FCB-Former	0.803	0.899	0.861	0.09	0.792	0.887	0.839	0.42
ESFPNet	0.793	0.891	0.849	< 0.05	0.794	0.892	0.843	0.51
Nanni’s Ens	0.806	0.903	0.863	0.32	0.795	0.891	0.843	0.56
HarDNet-DFUS	0.748	0.862	0.821	< 0.05	0.746	0.861	0.817	< 0.05
DET-Former	0.825	0.910	0.873	_	0.798	0.894	0.851	_

An article on polyp segmentation pointed out that it is difficult for a single network to make accurate predictions in many situations^20^. As shown in Table 2, excluding DET-Former and Nanni’s Ens, no single network consistently emerged as optimal across different datasets, reinforcing the challenge of achieving robust performance in colon polyp segmentation when faced with dataset variability. The models’ learning abilities were evaluated by training and testing them on identical datasets. In experiments where Kvasir and CVC-ClinicDB were used for training and testing, the performance metrics of DET-Former exceeded those of the comparator models, highlighting its superior learning capabilities. However, the results of statistical analyses show that DET-Former cannot significantly outperform some networks in terms of learning ability. DET-Former and Nanni’s Ens outperformed individual networks regarding *Dice*, *mDice* and *mIoU* metrics. These results suggest that the strategy of multi-model ensemble is expected to solve the problem of unstable learning ability of a single network on different data.

**Table 4:** The test results of the compare study in polypGen.

Train/Val	(C2, C3, C4, C5, C6) 90%/10%				(C1, C2, C4, C5, C6) 90%/10%				(C1, C2, C4, C5, C6) 90%/10%			
Test	C1				C2				C3			
Metric	Dice	mIoU	HD95	*p*	Dice	mIoU	HD95	*p*	Dice	mIoU	HD95	*p*
UNet	0.737	0.805	35.62	< 0.05	0.714	0.785	36.42	< 0.05	0.742	0.812	34.18	< 0.05
UperNet	0.818	0.853	23.05	< 0.05	0.806	0.832	28.54	< 0.05	0.820	0.856	21.64	< 0.05
Double-UNet	0.672	0.749	43.23	< 0.05	0.658	0.726	53.12	< 0.05	0.682	0.762	39.54	< 0.05
SS-Former	0.837	0.865	18.39	< 0.05	0.820	0.874	19.87	0.26	0.894	0.910	9.08	0.09
FCB-Former	0.848	0.875	17.65	< 0.05	0.803	0.862	23.32	0.08	0.902	0.916	9.06	0.20
ESFPNet	0.846	0.873	17.92	< 0.05	0.836	0.881	18.09	0.69	0.898	0.912	9.33	0.11
HarDNet-DFUS	0.831	0.867	18.82	< 0.05	0.813	0.869	21.67	0.19	0.852	0.879	12.61	< 0.05
DET-Former	0.872	0.894	12.91	_	0.843	0.886	17.37	_	0.912	0.922	7.63	_

**Table 5:** The test results of the compare study in polypGen.

Train/Val	(C1, C2, C3, C5, C6) 90%/10%				(C1, C2, C3, C4, C6) 90%/10%				(C1, C2, C3, C4, C5) 90%/10%			
Test	C4				C5				C6			
Metric	Dice	mIoU	HD95	*p*	Dice	mIoU	HD95	*p*	Dice	mIoU	HD95	*p*
UNet	0.491	0.697	69.50	< 0.05	0.520	0.701	58.23	< 0.05	0.713	0.798	33.70	< 0.05
UperNet	0.671	0.784	40.85	< 0.05	0.594	0.751	50.00	< 0.05	0.783	0.853	27.57	< 0.05
Double-UNet	0.367	0.615	99.86	< 0.05	0.509	0.677	59.68	< 0.05	0.617	0.733	50.81	< 0.05
SS-Former	0.597	0.750	53.72	< 0.05	0.672	0.790	38.09	0.23	0.835	0.883	17.74	0.85
FCB-Former	0.647	0.783	46.31	< 0.05	0.698	0.798	31.54	0.52	0.830	0.876	19.95	0.65
ESFPNet	0.616	0.763	51.40	< 0.05	0.645	0.774	40.99	< 0.05	0.836	0.885	12.76	0.98
HarDNet-DFUS	0.682	0.790	41.90	< 0.05	0.652	0.779	38.21	< 0.05	0.831	0.877	17.26	0.73
DET-Former	0.729	0.826	33.04	_	0.710	0.809	31.19	_	0.843	0.886	19.38	_

**Table 6:** The results of comparison with other weight optimization algorithms in CVC-ClinicDB.

Ensemble strategy	CVC-ClinicDB			
	Weight	Dice	mIoU	*p*
Average weight	[0.17, 0.17, 0.17, 0.17, 0.17, 0.17]	0.952	0.952	0.95
Base PSO	[0.21, 0.09, 0.17, 0.34, 0.00, 0.19]	0.951	0.951	0.90
Base CS–PSO	[0.34, 0.23, 0.43, − 0.14, 0.45, 0.26]	0.951	0.952	0.93
Base RHB–PSO	[0.28, 0.22, − 0.10, 0.24, 0.36, 0.04]	0.951	0.950	0.83
Our	[0.16, 0.03, 0.46, _ , 0.44, 0.05]	0.953	0.953	_

**Table 7:** The results of comparison with other weight optimization algorithms in Kvasir.

Ensemble strategy	Kvasir			
	Weight	*Dice*	*mIoU*	*p*
Average weight	[0.17, 0.17, 0.17, 0.17, 0.17, 0.17]	0.908	0.909	0.19
Base PSO	[0.23, 0.60, − 0.15, 0.85, 0.08, − 0.61]	0.910	0.911	0.25
Base CS–PSO	[0.44, 0.97, 0.06, 1.20, − 1.08, − 0.74]	0.907	0.908	0.16
Base RHB–PSO	[− 0.32, 0.85, 0.01, 0.52, 0.21, − 0.28]	0.904	0.907	0.13
Our	[0.39, 0.22, − 0.21, 0.68, _ , − 0.15]	0.923	0.924	_

**Figure 4:**
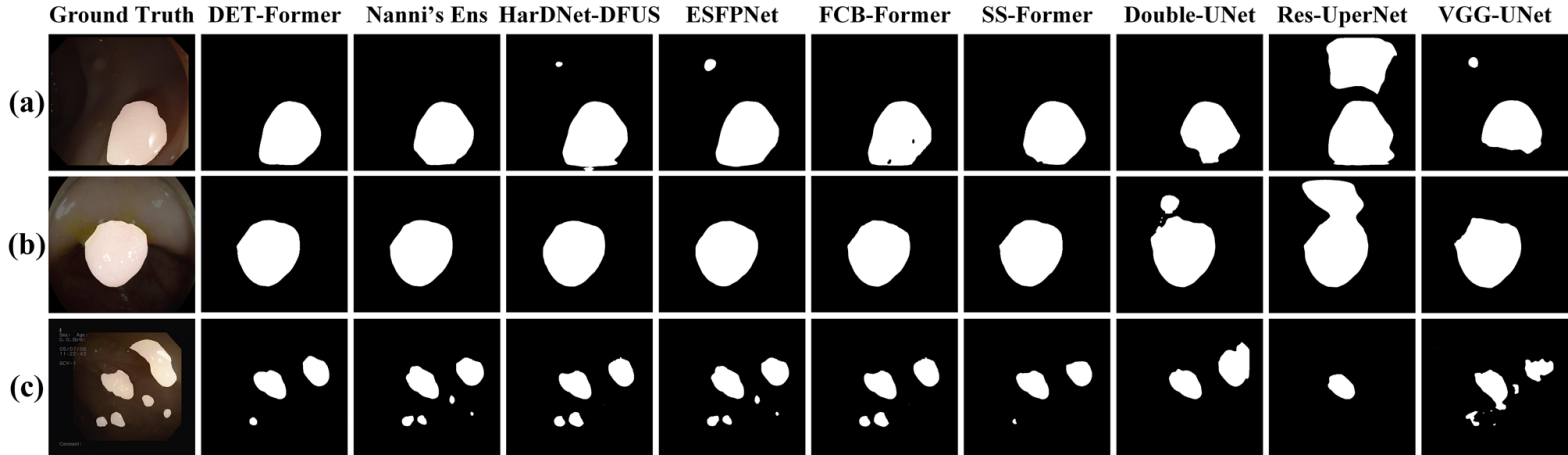
Visual segmentation results. (From top to bottom, there are CVC-ColonDB, ETIS-LaribPolypDB, and PolypGen datasets).

As shown in Table 3, DET-Former’s evaluation metrics - *Dice*, *mDice* and *mIoU* - show superiority over other models on the ETIS-LaribPolypDB and CVC-ColonDB datasets, suggesting superior generalisation ability. Statistical analysis reveals that DET-Former significantly outperforms other models on the ETIS-LaribPolypDB dataset, except FCB-Former and Nanni’s Ensemble. However, this significant advantage is not evident when analysing the CVC-ColonDB dataset. Among them, CVC-ClinicDB and CVC-ColonDB were used to form CVC-EndoSceneStill^68^ in the MICCAI2015 challenge^69^. The significant difference between DET-Former and the comparison model is larger in ETIS-LaribPolypDB and CVC-ColonDB. To better analyse whether the generalisation ability of DET-Former can be significant due to the comparison model, we performed generalisation experiments on the multi-centre PolypGen.

Tables 4 and 5 shows the performance of DET-Former on the PolypGen multi-centre dataset. DET-Former shows a significant improvement over competing models in centres C1 and C4. However, its performance advantage is less pronounced in centres C2, C3, C5 and C6, where it outperforms only some models. Although DET-Former exhibits superior generalisation capabilities on the multi-centre PolypGen dataset, it does not achieve significant dominance in all centres. To further investigate the limitations of DET-Former’s generalisation ability in certain centres, we analyse this in conjunction with the visual segmentation results in Fig 4. Figure 4c shows a decrease in the segmentation accuracy of DET-Former, especially in cases where different networks produce different false-negative segmentations. This problem arises because the DET-Former ensemble has multiple sub-models, and the false negatives from these sub-models are variable, especially in complex scenarios such as the independent distribution of multiple polyps. The complexity of these divergent false-negative segmentations poses a significant challenge for ensemble learning in colon polyp segmentation. Conversely, false-positive segmentations are less frequent and tend to occur sporadically across models, as shown in Fig. 4a, b. DET-Former is an ensemble learning structure that can optimise the false-positive results of individual false-positive segmentation models through multiple sub-models. Thus, ensemble learning has advantages in false-positive segmentation. In the future, more effective balancing of false-negative and false-positive results in ensemble learning needs to be achieved to address the problem of false negatives in colon polyp segmentation.

### Model ensemble experiment

In the model ensemble experiments, the optimal sub-model of the previous compare experiments of DET-Former on Kvasir and CVC-ClinicDB datasets is chosen as the sub-model of the model ensemble experiments. Then to measure the effectiveness of the improvement, we choose the RBH-PSO algorithm and CSPSO algorithm, which are the closest to SDBH-PSO, as well as the classical PSO algorithm and weight averaging as the BASELINE algorithm to adjust the weights of the ensemble model. The weight optimization results are shown in Table 6.

As shown in Table 6, under the CVC-ClinicDB data, the results of the outputs of various strategies are basically the same, and it can be seen that the method of improving the ensemble effect through weight optimization is not applicable to all cases. Even so, by comparing Table 2, it can be seen that compared with single network segmentation, the ensemble learning improves the segmentation accuracy greatly. As shown in Table 7, It is worth noting that the superiority of PSO over RBH-PSO and CSPSO under the Kvasir dataset also indicates that not all of the proposed improved algorithms based on the PSO algorithm are well suited for the ensemble task of the colon polyp segmentation network. While our ensemble model does not demonstrate statistically significant superiority in performance compared to the comparison method, the ensemble model of our method eliminates the sub-models and improves the performance instead of degrading it. Despite utilizing only five-sixths of the model usage compared to the comparative methods, our method maintains performance levels. This suggests that the proposed SDBH-PSO Ensemble based on the polyp segmentation task is better than PSO weight optimization with average weights, which verifies that our improvement is suitable for this task.

**Figure 5:**
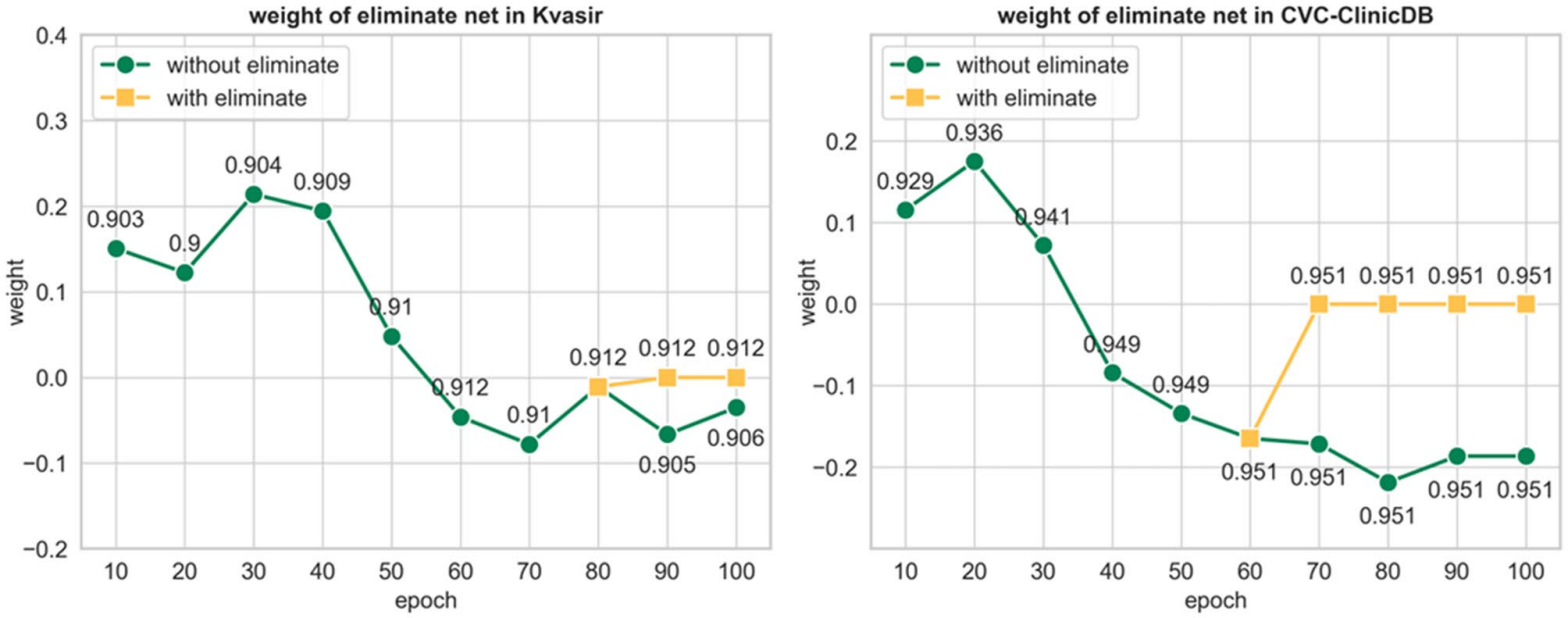
The green line indicates the ensemble weights of the sub-models retained during training under the SDBH-PSO calculation. The yellow line indicates that the training was stopped after setting the sub-model weights to zero, where the mean intersection and union set (*mIoU*) of the real-time ensemble models are shown around the corresponding points.

To further explore the effectiveness of the sub-modeling strategy of SDBH-PSO Ensemble eliminated sub-models, we engage the eliminated sub-models in real-time ensemble, and their ensemble weights computed by SDBH-PSO for every ten epochs are shown in Fig. 5.

As shown in Tables 6 and 7, the 5th sub-model is eliminated on the Kvasir dataset and the 4th sub-model is eliminated on the CVC-ClinicDB dataset. As shown in Fig. 5, the sub-model eliminated on the Kvasir dataset is eliminated by the SDBH-PSO Ensemble strategy at epoch 60. The sub-model eliminated on the CVC-ClinicDB dataset is eliminated at epoch 70. On the Kvasir dataset, SDBH-PSO Ensemble improved performance by filtering out models that were not suitable for ensemble. On the CVC-ClinicDB dataset, the elimination of the sub-models filtered out by the SDBH-PSO Ensemble does not improve the performance, but also does not degrade the overall performance.

The experiments verify the ability of SDBH-PSO Ensemble to select sub-models and the effectiveness of sub-model selection by eliminating sub-model strategy. A review article pointed out that model selection is a major challenge for ensemble learning^30^. It is believed that the ensemble method is not only applicable to colon polyp segmentation, but also can realize adaptive sub-model selection for different datasets by pairing with suitable sub-models in other tasks.

### Feature fusion experiments

**Figure 6:**
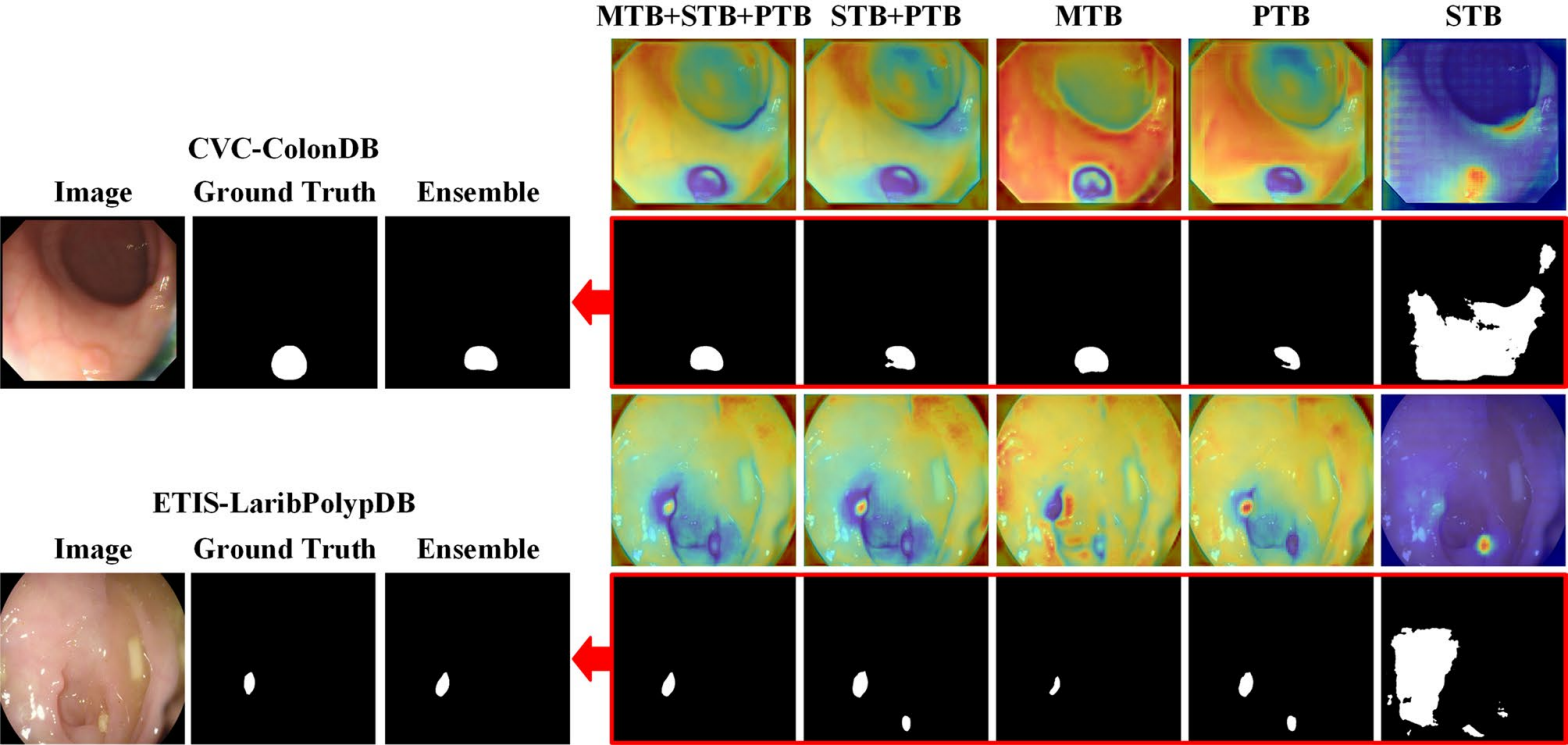
Feature heat maps of the five branches with the segmentation results of the corresponding branches. The results after multi-output ensemble by MHC Ensemble are shown as red clippings pointing to.

In the feature fusion experiments, since the ability of feature extraction and fusion can be better demonstrated on datasets that have never been involved in training, we train and validate on Kvasir and CVC-ClinicDB datasets and test on CVC-ColonDB and ETIS-LaribPolypDB datasets, and the results of the tests are shown in Fig. 6 and Table 8.

**Table 8:** The performance of different branches in Treble-Former.

Output head	CVC-ColonDB			ETIS-LaribPolypDB		
	Dice	mIoU	*p*	Dice	mIoU	*p*
STB	0.632	0.713	<0.05	0.540	0.640	<0.05
PTB	0.776	0.833	0.66	0.796	0.856	<0.05
MTB	0.785	0.836	0.76	0.753	0.814	<0.05
STB+PTB	0.783	0.835	0.72	0.802	0.857	0.08
STB+PTB+MTB	0.786	0.837	0.79	0.807	0.858	0.17
MHC ensemble	0.794	0.843	_	0.821	0.868	_

As shown in Table 8, the outputs of STB, PTB and MTB after fusion are better for STB+PTB and STB+PTB+MTB than STB, PTB and MTB before fusion, and the segmentation performance is further improved after integration. As shown in Fig. 6, the visualization of the features by heat map shows that the features extracted from different branches are quite different, and along with the feature fusion, the output results appear to be improved accordingly. Our ensemble strategy successfully compensates for the different branch segmentation defects. In this case, Treble-Former is used to fuse multiple branches and ensemble multiple output heads through MHC Ensemble, so the ensemble output results are the output results of Treble-Former.

Through Fig. 6 and Table 8, it can be seen that no branch in STB, PTB and MTB can perform the segmentation ability stably, but through feature fusion, the boundary features of the polyps become more obvious, which further compensates for the deficiency of some branches after weighted ensemble. Statistical analyses reveal that the MHC Ensemble significantly surpasses the branch STB only after the fusion of multi-branch advanced features on the CVC-ColonDB dataset. However, it significantly outperforms all three branches prior to fusion on the ETIS-LaribPolypDB dataset. These findings underscore the MHC Ensemble’s capacity to leverage advanced semantic features effectively. High-level semantic features are more appropriate for the model to perform better^12^. Our research confirms that this improvement extends to colon polyp segmentation. As artificial intelligence progresses, we anticipate the introduction of more sophisticated backbones and networks that will surpass the performance of current models such as PvTv2, Mix Transformer, Double UNet, and FCB-Former. The MHC Ensemble, which performs layer-by-layer fusion of advanced semantic features on them, will continue to exist as a reference value.

## Conclusion

In this study, we propose a novel Dual Ensemble System with Treble Transformer (DET-Former). The system first constructs a multi-branch ensemble network Treble-Former with three different Transformers. Then, to improve the stability under different datasets, we propose DET-Former with SDBH-PSO Ensemble structure. Among them, the Treble-Former’s approach, which employs a multi-branch, layer-by-layer fusion of high-level semantic features, represents a promising direction for developing more accurate segmentation models in the future. Meanwhile, DET-Former maintains stable, high-performance segmentation relative to other networks, suggesting that ensemble learning is expected to solve the problem of unstable performance of a single network on different colon polyp datasets. In addition, experimental evidence shows that the SDBH-PSO ensemble can adaptively select sub-models during training, providing valuable insights into model selection for ensemble learning.

## Data Availability

The datasets used in this study are publicly available at: Kvasir-SEG: https://datasets.simula.no/kvasir-seg/. CVC-ClinicDB: https://polyp.grand-challenge.org/CVCClinicDB/. ETIS-LaribpolypDB: https://drive.google.com/drive/folders/10QXjxBJqCf7PAXqbDvoceWmZ-qF07tFi?usp=share_link. CVC-ColonDB: https://drive.google.com/drive/folders/1-gZUo1dgsdcWxSdXV9OAPmtGEbwZMfDY?usp=share_link.

## Code availability

We want to clarify that all codes employed for data analysis, including training, validation, testing, and the trained colonic polyp segmentation networks, have been separately at https://github.com/xucuncun/Dual-Ensemble-System-with-Treble-Former and https://github.com/xucuncun/Treble-Former.
